# Therapeutic Potential of Chimeric Antigen Receptor-Expressing Mesenchymal Stem Cells in the Treatment of Inflammatory and Autoimmune Diseases

**DOI:** 10.3390/ijms26167795

**Published:** 2025-08-12

**Authors:** Vladislav Volarevic, Carl Randall Harrell, Crissy Fellabaum, Valentin Djonov, Ana Volarevic

**Affiliations:** 1 Departments of Genetics, Microbiology and Immunology, Center for Research on Harmful Effects of Biological and Chemical Hazards, Faculty of Medical Sciences, University of Kragujevac, 69 Svetozara Markovica Street, 34000 Kragujevac, Serbia; 2 Regenerative Processing Plant, LLC, US Highway 19 N, Palm Harbor, FL 34176, USA; crissy@regenerativeplant.org; 3 Institute of Anatomy, University of Bern, Baltzerstrasse 2, 3012 Bern, Switzerland; valentin.djonov@unibe.ch; 4 Departments of Psychology, Center for Research on Harmful Effects of Biological and Chemical Hazards, Faculty of Medical Sciences, University of Kragujevac, 69 Svetozara Markovica Street, 34000 Kragujevac, Serbia; ana.volarevic@fmn.kg.ac.rs

**Keywords:** chimeric antigen receptor, mesenchymal stem cells, autoimmune diseases, inflammatory diseases, cell-based therapy

## Abstract

Chimeric antigen receptor-engineered mesenchymal stem cells (CAR-MSCs) represent a novel and highly adaptable platform for the targeted treatment of inflammatory and autoimmune diseases. By integrating the inflammation-homing and immunomodulatory properties of mesenchymal stem cells (MSCs) with the antigen-specific recognition and activation potential of chimeric antigen receptors (CARs), CAR-MSCs enable site-specific delivery of therapeutic agents directly to inflamed or diseased tissues. This dual functionality enhances therapeutic precision while minimizing off-target effects and systemic toxicity. Recent preclinical studies have demonstrated the efficacy of CAR-MSCs in modulating pathogenic immune responses, reducing local inflammation, and promoting tissue repair in various disease models. CAR-MSCs have been engineered to recognize and interact with disease-specific antigens or inflammatory markers, allowing them to selectively suppress the activation and proliferation of autoreactive immune cells. This targeted immunosuppression offers a promising strategy for restoring immune tolerance without the risks associated with systemic immunosuppression. In this review, we provide a comprehensive overview of recent developments in CAR-MSC design, highlight mechanisms by which CARs enhance MSC functionality, and discuss key challenges, including safety, scalability, and regulatory considerations. Collectively, these emerging approaches hold substantial promise for reshaping future therapies for inflammatory and autoimmune diseases.

## 1. Introduction

Mesenchymal stem cells (MSCs) have emerged as a pivotal component in the field of regenerative medicine, owing to their multipotent differentiation potential and robust immunomodulatory capabilities [1,2,3]. These adult stem cells, originally derived from mesodermal tissues, are capable of differentiating into mesodermal lineage cells, including osteoblasts, chondrocytes, and adipocytes [1,2]. In addition to this classical mesodermal commitment, accumulating evidence indicates that MSCs, when cultured under specific induction conditions, possess the plasticity to transdifferentiate into cells of ectodermal and endodermal origin, broadening their potential application in diverse tissue types [1,2].

Beyond their differentiation capabilities, MSCs are widely recognized for their profound immunomodulatory properties, which position them as promising therapeutic agents in the management of various inflammatory and autoimmune disorders [3,4]. MSCs can interact with a broad range of immune cells, including innate immune effectors such as macrophages, dendritic cells (DCs), neutrophils, natural killer (NK) cells, and natural killer T (NKT) cells, as well as adaptive immune cells such as T and B lymphocytes [3,4]. MSCs may modulate phenotype and function of immune cells in both juxtacrine (direct cell–cell contact) and paracrine mechanisms, through the activity of MSC-derived immunoregulatory factors [4,5]. These secreted molecules include cytokines and soluble mediators such as transforming growth factor-β (TGF-β), prostaglandin E2 (PGE2), indoleamine 2,3-dioxygenase (IDO), interleukin-10 (IL-10), nitric oxide (NO), hepatocyte growth factor (HGF), and regulatory microRNAs (miRNAs), all of which contribute to the immunosuppressive profile of MSCs [5]. Through these mechanisms, MSCs can inhibit the proliferation and effector functions of T cells, B cells, NK cells, and NKT cells; promote the expansion and functional activation of immunosuppressive regulatory T cells (Tregs); induce polarization of macrophages from a pro-inflammatory (M1) toward an anti-inflammatory (M2) phenotype; and suppress the maturation, migration, and antigen-presenting function of DCs [3,4,5].

Importantly, the immunomodulatory effects of MSCs are context-dependent and are significantly influenced by the surrounding inflammatory milieu [5]. MSCs become activated, or “licensed,” in response to inflammatory cytokines such as interferon-γ (IFN-γ), tumor necrosis factor-α (TNF-α), and interleukin-1β (IL-1β), which are typically elevated in damaged tissues [5]. This inflammation-induced licensing enhances the immunoregulatory functions of MSCs, allowing them to respond adaptively to the pathological environment. According to their immunomodulatory properties, MSCs have been categorized into two distinct phenotypic states: a pro-inflammatory (MSC1) and an anti-inflammatory, immunosuppressive (MSC2) phenotype [6]. Importantly, this binary classification reflects the dual immunomodulatory potential of MSCs and is not a rigid distinction. MSCs exhibit significant phenotypic plasticity, dynamically shifting between MSC1 and MSC2 states in response to microenvironmental cues, including cytokines, adipokines, alarmins, and pathogen-associated molecular patterns. These external stimuli critically shape MSC behavior and determine whether they adopt a pro- or anti-inflammatory functional profile [6]. Under conditions of acute inflammation, characterized by elevated concentrations of inflammatory cytokines (TNF-α, IFN-γ, IL-1β), MSCs are typically driven toward the MSC2 phenotype, marked by enhanced secretion of immunosuppressive mediators and suppression of effector immune cell responses [6]. This phenotype plays a key role in promoting tissue repair and restoring immune homeostasis. In contrast, in the context of chronic, low-grade inflammation, which is commonly observed in the adipose tissue of individuals with obesity, MSCs undergo “licensing” toward a pro-inflammatory MSC1 phenotype. This shift is promoted by sustained exposure to moderate levels of inflammatory cytokines (TNF-α, IL-1β), adipokines (leptin, resistin), and other inflammatory stimuli that accumulate in the obese adipose tissue microenvironment [6]. MSC1 polarization results in a transcriptional and secretory program characterized by elevated production of pro-inflammatory cytokines and chemokines, including IL-6, IL-8, and monocyte chemoattractant protein-1 (MCP-1). These molecules facilitate the recruitment and activation of various immune cells, notably Th17 and Th22 cells, γδ T cells, and macrophages. Importantly, when MSCs adopt the MSC1 phenotype, they undergo a functional shift away from their inherent immunosuppressive properties. MSC1 cells actively propagate a pro-inflammatory milieu, perpetuating tissue inflammation and immune cell infiltration [6]. By sustaining an inflammatory niche, MSC1 cells significantly contribute to systemic immune activation, including epigenetic reprogramming of immune cells that favors Th1/Th17 polarization. As a result, MSC1 activity is increasingly implicated in the progression of Th1 and Th17 cell-driven chronic inflammatory and autoimmune diseases [6].

Importantly, MSCs have garnered significant interest as a cellular therapeutic platform due to their low immunogenicity, which enables their administration across allogeneic barriers with minimal risk of immune rejection [7]. Unlike other cell types, MSCs lack the expression of major histocompatibility complex (MHC) class II and co-stimulatory molecules (CD40, CD80, and CD86) under basal conditions, rendering them poorly immunogenic and reducing the likelihood of activating host T cell responses [8]. This unique immunological profile allows MSCs to evade immune surveillance and persist transiently in the host tissue, where they can exert their therapeutic effects without eliciting robust alloimmune reactions [9]. A large number of experimental studies demonstrated that MSCs efficiently alleviated immune cell-driven inflammatory conditions (inflammatory bowel disease, chronic obstructive pulmonary disease, graft-versus-host disease (GvHD), type 1 diabetes), where sustained inflammation and immune dysregulation contribute to disease progression [9,10,11].

Despite extensive preclinical evidence supporting the therapeutic potential of MSCs in the treatment of inflammatory conditions, the clinical translation of MSC-based therapies remains significantly constrained by several critical limitations [12,13,14]. The tissue of MSCs’ origin significantly influences their biological properties and, consequently, their therapeutic potential. MSCs can be isolated from various sources, including bone marrow, adipose tissue, umbilical cord, placenta, dental pulp, and synovial membrane, each conferring distinct phenotypic, functional, and molecular characteristics [15]. For instance, bone marrow-derived MSCs (BM-MSCs) typically exhibit robust osteogenic differentiation capacity and strong immunomodulatory functions, making them suitable for bone repair and immune-mediated disorders [15]. In contrast, adipose-derived MSCs (AT-MSCs) are more abundant, easier to harvest, and display superior proliferative capacity, though they may possess a more pro-inflammatory secretome, particularly in individuals with obesity or metabolic syndrome [15]. Umbilical cord-derived MSCs (UC-MSCs), obtained from neonatal tissues, exhibit higher proliferative rates, longer telomeres, and a more primitive phenotype with lower immunogenicity, favoring their use in allogeneic transplantation [15]. The tissue microenvironment imprints MSCs with distinct epigenetic and transcriptional profiles that influence their secretion of cytokines, growth factors, and extracellular vesicles, as well as their interactions with immune and stromal cells [16]. Furthermore, disease-related alterations in the tissue niche, such as chronic inflammation, hypoxia, or oxidative stress, can induce senescence or shift MSCs toward a dysfunctional, pro-inflammatory MSC1 phenotype, reducing therapeutic efficacy. Thus, both the anatomical source and the physiological or pathological condition of the donor tissue are critical determinants of MSC quality, safety, and therapeutic effectiveness [17].

MSC-based therapies employ either autologous MSCs, derived from the patient’s own tissues, or allogeneic MSCs, obtained from genetically unrelated donors [6,18]. Each approach offers distinct advantages and limitations that must be critically evaluated in the context of clinical application. Autologous MSCs present a clear immunological advantage due to their histocompatibility, thereby minimizing the risk of immune rejection and obviating the need for immunosuppressive regimens. Additionally, autologous transplantation raises fewer ethical concerns and may support personalized treatment strategies [18]. However, MSCs isolated from individuals with advanced age or underlying pathological conditions such as metabolic syndrome, type 2 diabetes, or chronic inflammatory states often exhibit impaired proliferative capacity, increased cellular senescence, reduced differentiation potential, and a skewing toward a pro-inflammatory MSC1 phenotype [19,20]. These alterations can compromise their therapeutic efficacy. Furthermore, the procurement and ex vivo expansion of autologous MSCs is time-intensive and technically demanding, limiting their feasibility for acute or time-sensitive indications [6]. Allogeneic MSCs offer several practical and logistical advantages. Cells can be isolated from healthy young donors with optimal regenerative profiles, expanded in large batches under controlled conditions, and cryopreserved for immediate, on-demand use. This enables the generation of standardized, quality-assured cell products suitable for broad clinical distribution [18]. Owing to their low expression of major histocompatibility complex (MHC) class II and co-stimulatory molecules, MSCs are considered relatively immune evasive and have been employed in allogeneic settings with acceptable safety profiles in numerous clinical trials [6]. Nonetheless, repeated or high-dose administration may lead to alloimmune recognition, immune-mediated clearance, and reduced persistence in vivo, potentially diminishing therapeutic benefit. Moreover, concerns remain regarding the long-term immunological consequences of allogeneic MSC transplantation, particularly in sensitized recipients [6]. A critical aspect of allogeneic MSC-based therapy is the rigorous selection of donors. MSCs derived from individuals affected by chronic inflammatory, autoimmune, or oncologic diseases may exhibit a pathogenic, pro-inflammatory MSC1 profile, characterized by elevated production of inflammatory cytokines and chemokines, which can perpetuate immune cell recruitment and exacerbate tissue inflammation. The use of such cells in therapeutic contexts poses a significant risk of aggravating, rather than ameliorating, disease processes [18]. Consequently, donor screening protocols must exclude individuals with these pathological conditions, and robust phenotypic and functional characterization of MSC preparations should be implemented to ensure the selection of cells with anti-inflammatory and immunoregulatory properties consistent with the MSC2 phenotype [6].

Another important obstacle for successful MSC-based therapy is the inherently low homing efficiency of systemically administered MSCs to the sites of tissue injury or inflammation. After intravenous delivery, only a small fraction of MSCs successfully migrates to the intended target tissue, with the majority becoming sequestered in non-target organs such as the lungs, liver, and spleen, thereby diminishing therapeutic efficacy [13]. Another major limitation is the lack of intrinsic targeting specificity within MSC populations [21]. Although MSCs possess some natural tropism for inflamed or damaged tissues, they do not possess precise homing mechanisms capable of navigating the complex architecture and cellular heterogeneity of diseased microenvironments [21]. As a result, their capacity to deliver therapeutic agents with spatial and temporal precision remains suboptimal [13,14]. These challenges underscore the need for advanced bioengineering strategies to enhance MSC homing, survival, and functional specificity [13,14].

In recent years, genetic engineering has emerged as an innovative strategy for augmenting the therapeutic efficacy of MSCs [22,23,24]. Among the various genetic modification approaches, the integration of chimeric antigen receptor (CAR) technology, originally developed to enhance the tumor-targeting capabilities of T cells, has shown particular promise when applied to MSCs [22,23]. CARs are synthetic, modular receptors that combine an antigen-recognition domain, typically derived from a monoclonal antibody, with intracellular signaling motifs that mediate cell activation. Importantly, CARs enable antigen recognition in a non-MHC-restricted manner, thereby allowing engineered cells to recognize and bind specific surface antigens with high affinity and selectivity, independent of traditional antigen presentation pathways [22,23]. When introduced into MSCs, CAR constructs can significantly enhance the functional attributes of these cells. Specifically, CAR-expressing MSCs (CAR-MSCs) can be designed to recognize disease-associated antigens, thereby improving their ability to home to pathological tissues, retain at the site of disease, and deliver therapeutic payloads with increased spatial precision [22]. In addition to enhanced targeting, CAR-MSCs can be further engineered to secrete immunomodulatory cytokines or other bioactive molecules in a regulated, antigen-dependent manner, thereby expanding their therapeutic repertoire and enabling more controlled and context-specific intervention [22,24]. By redirecting MSC activity toward specific cellular targets and enhancing their functional persistence within hostile microenvironments, CAR engineering holds the potential to overcome many of the limitations currently associated with MSC-based therapy [22,24].

Accordingly, in this review paper, we summarize recent advances in the development of CAR-engineered MSCs, explore the underlying mechanisms through which CARs augment the function of MSCs, and discuss current challenges and future perspectives of CAR-MSC-based therapy. An extensive literature review was carried out in June 2025 across several databases (MEDLINE, EMBASE, and Google Scholar), from 1990 to the present. Key words used in the selection were “mesenchymal stem cells”, “chimeric antigen receptor”, “immunomodulation”, “autoimmune diseases”, “inflammatory diseases”, and “therapy”. All journals were considered, and an initial search retrieved 128 articles. The abstracts of all these articles were subsequently reviewed by two of the authors (VV and CRH) independently to check their relevance to the subject of this manuscript. Eligible studies had to delineate the molecular and cellular mechanisms that are responsible for the therapeutic potential of CAR-expressing MSCs in the treatment of autoimmune and inflammatory diseases, and their findings are analyzed in this review.

## 2. Development, Phenotype, and Function of CAR-Expressing MSCs

CAR-MSCs typically involve the introduction of a CAR gene construct into MSCs using either viral or non-viral gene delivery methods [22,23,24,25]. Among viral vectors, lentiviral and retroviral systems are most commonly employed [22]. Lentiviral vectors are particularly advantageous for MSC transduction due to their ability to efficiently transduce both dividing and non-dividing cells, combined with relatively low immunogenicity and stable gene integration [22]. In this approach, the CAR transgene is cloned into a viral expression plasmid under the control of a constitutive or inducible promoter. This plasmid is then used to produce viral particles in packaging cell lines, followed by transduction of cultured MSCs with the viral supernatant [22,23].

Non-viral methods, such as electroporation, nucleofection, and transposon-based systems, have also been employed to introduce CAR constructs into MSCs [22]. These techniques reduce the risk of insertional mutagenesis associated with integrating viral vectors; however, they are generally associated with lower transfection efficiency and transient CAR expression, which may limit long-term therapeutic efficacy [22]. Following gene transfer, CAR-MSCs undergo rigorous characterization to confirm appropriate CAR expression, retention of MSC identity, and acquisition of desired functional properties [22,24]. Structurally, a CAR molecule comprises three principal domains: (i) an extracellular antigen-recognition domain, typically composed of a single-chain variable fragment (scFv) derived from a monoclonal antibody that binds specifically to a disease-associated surface antigen; (ii) a hinge/transmembrane domain that anchors the receptor within the MSC plasma membrane and contributes to structural stability; (iii) one or more intracellular signaling domains, which are responsible for mediating downstream cellular responses such as enhanced migration, survival, or secretion of therapeutic factors in response to antigen engagement [22,24].

**Figure 1:**
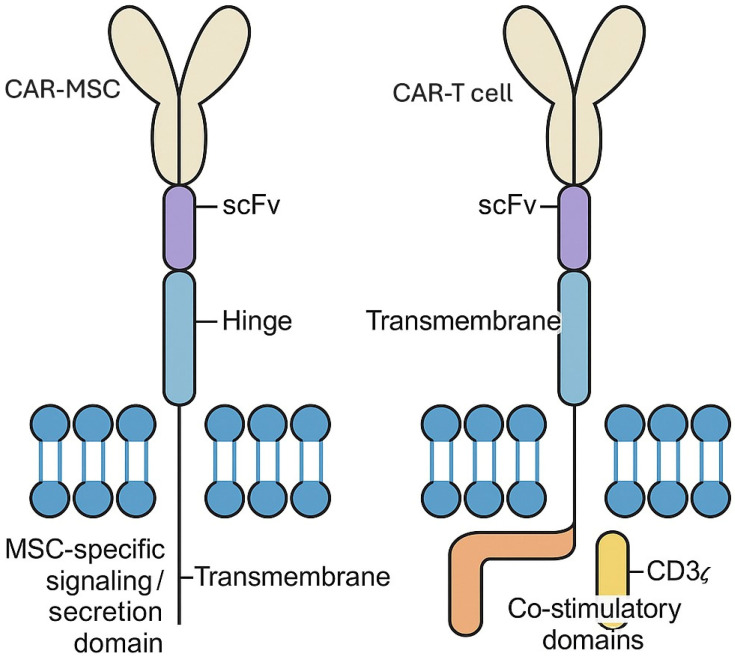
Structural differences between CAR receptors in CAR-MSCs and CAR-T cells. The schematic illustrates the typical architecture of chimeric antigen receptors (CARs) expressed on CAR-MSCs and CAR-T cells. Both receptor types contain an extracellular single-chain variable fragment (scFv) for antigen recognition and a hinge and transmembrane domains. CAR-T cells typically incorporate intracellular signaling domains derived from CD3ζ along with one or more co-stimulatory domains (CD28 or 4-1BB) to promote T cell activation, proliferation, and cytotoxic function. In contrast, CARs used in CAR-MSCs often feature modified or attenuated intracellular domains to enable antigen-specific homing or secretion of therapeutic payloads without inducing immune effector functions. Structural differences reflect the distinct biological roles and therapeutic strategies associated with each CAR-modified cell type.

In a manner similar to how it is designed in CAR-T cells, the scFv in CAR-MSC is usually linked to the rest of the CAR structure via a flexible hinge or spacer region, which provides spatial flexibility necessary for effective target binding, particularly in complex tissue environments [22,24]. However, the main difference between CAR-MSCs and CAR-T cells is that CAR-T cells are engineered for immune activation and cytotoxicity, while CAR-MSCs are designed for targeted delivery or therapeutic secretion without triggering immune responses. Therefore, structurally, CAR-T receptors, in addition to the scFv domain and transmembrane region, contain an intracellular signaling region that includes CD3ζ and co-stimulatory domains (CD28 or 4-1BB), enabling full T cell activation. In contrast, CARs in MSCs often lack these potent intracellular signaling regions or use truncated or alternative intracellular motifs to avoid triggering immune effector functions, reflecting their immunomodulatory role [22,24] (Figure 1).

Surface expression of CARs on engineered MSCs is typically verified using flow cytometry, employing either antigen-binding assays or tag-based detection systems [22,25]. Additional analyses such as reverse transcription quantitative PCR (RT-qPCR) and Western blotting are performed to assess CAR transcription and translation, respectively. Crucially, CAR-MSCs must retain their defining phenotypic characteristics, including the expression of canonical surface markers CD73, CD90, and CD105, as well as their multipotent differentiation potential. These features are routinely evaluated through immunophenotyping and tri-lineage differentiation (adipogenic, osteogenic, and chondrogenic) assays [22,25].

Functional validation of CAR-MSCs includes in vitro co-culture assays with antigen-expressing target cells to assess antigen-specific migratory responses, cytokine production, or delivery of therapeutic molecules [22,25]. Once MSC identity, CAR expression, and functional competence are confirmed, CAR-MSCs are expanded under optimized in vitro culture conditions that preserve their stemness, viability, and therapeutic functionality for downstream clinical or experimental applications [22,25].

## 3. Therapeutic Potential of CAR-Expressing MSCs in the Treatment of SLE

SLE is a chronic, multifactorial autoimmune disorder characterized by the loss of immunological tolerance to self-antigens, resulting in the production of pathogenic autoantibodies and the formation of immune complexes that contribute to widespread inflammation and multi-organ damage [26]. The immunopathogenesis of SLE involves aberrant activation of autoreactive B and T lymphocytes, dysregulated cytokine networks (elevated levels of type I interferons, IL-6, and B cell-activating factor (BAFF)), and impaired clearance of apoptotic debris, all of which contribute to sustained autoimmunity and tissue injury [27]. Current therapeutic strategies, including corticosteroids, antimalarials, immunosuppressive agents, and biologics targeting B cells, offer symptomatic relief and disease control in many SLE patients but are often associated with systemic immunosuppression, increased risk of infections, and limited long-term efficacy in refractory cases [28]. In this context, MSCs have emerged as a promising therapeutic modality due to their potent immunomodulatory properties, including the ability to suppress autoreactive immune cell activation, promote expansion of Tregs, and modulate pro-inflammatory cytokine responses [29]. However, despite encouraging preclinical and early clinical data, the therapeutic efficacy of MSCs remains inconsistent, largely due to challenges such as poor homing to inflamed tissues, limited persistence and survival in hostile microenvironments, and lack of antigen specificity [29]. These limitations underscore the need for advanced strategies such as genetic engineering of CAR-MSCs, which would have enhanced targeting capabilities and functional potency for the treatment of SLE and other autoimmune diseases [30].

For the purpose of immunosuppressive therapy, CAR-MSCs could be strategically designed to recognize and respond to inflammatory signals or disease-specific antigens present in particular autoimmune diseases [22,23,24,25]. In line with these findings, Zhao and colleagues enhanced the therapeutic potential of MSCs for SLE treatment by equipping them with CARs specific for CD19, a surface antigen expressed on B cells [31]. The engineered CAR construct (designated as “CAR1”) incorporated a CD19-specific scFv fused to the intracellular domain of the IFN-γ receptor [31]. This design aimed to couple recognition of B cell-specific antigen (CD19) to the activation of the IFN-γ receptor downstream JAK2/STAT1 signaling pathway, which is a key axis in MSC-mediated immunosuppression [5,31]. To identify the most effective construct, multiple CAR variants were evaluated for their ability to induce phosphorylation of Janus kinase 2 (JAK2) and expression of immunoregulatory IDO, both at baseline and upon antigen stimulation [5,31]. MSCs were transduced with viral vectors encoding CAR constructs and were subsequently evaluated for CAR expression, antigen binding, and retention of phenotypic identity [31]. Immunofluorescence staining demonstrated specific binding of CAR1-MSCs to CD19 antigens [31]. Functional activation of the engineered cells was validated using in vitro stimulation assays with recombinant CD19 protein and CD19^+^ B cells, followed by Western blot analysis to detect downstream JAK2/STAT1 activation and IDO expression [31].

CAR1-MSCs exhibited robust and antigen-specific activation of the JAK2/Signal Transducer and Activator of Transcription 1 (STAT1) pathway, accompanied by up-regulated IDO expression, upon engagement with CD19^+^ target cells [31]. These findings confirmed the functionality of the synthetic CAR design in triggering downstream immunosuppressive signaling cascades [31]. Importantly, CAR1-MSCs retained typical mesenchymal features and did not exhibit changes in surface marker expression following genetic modification. To assess the immunomodulatory function of CAR1-MSCs, human peripheral blood mononuclear cells (PBMCs) were co-cultured with either unmodified MSCs or CAR1-MSCs [31]. Flow cytometric analysis revealed that both cell types effectively inhibited the activation of CD3^+^ T cells. However, CAR1-MSCs demonstrated significantly greater suppression of activated CD19^+^ B cells compared to their unmodified counterparts [31]. This selective enhancement in B cell immunosuppression was attributed to the antigen-specific, CD19-dependent activation of the engineered CAR1 construct and the subsequent up-regulation of IDO. The findings of this study demonstrate that MSCs can be successfully engineered to express a functional CAR targeting CD19, thereby enabling them to exert targeted, antigen-specific immunosuppressive effects [31]. The CAR1-MSCs selectively inhibited the activation of B cells without compromising MSC identity. By integrating CAR technology with the intrinsic immunomodulatory properties of MSCs, this approach offers a promising strategy for the treatment of SLE and other B cell-mediated autoimmune disorders, providing a strong preclinical rationale for further investigation of CAR-MSC platforms as precision immunotherapies in autoimmune and inflammatory disease contexts [31].

## 4. CAR-MSCs as Potentially Novel Therapeutic Agents for the Treatment of GvHD

GvHD is a severe and potentially life-threatening complication following allogeneic hematopoietic stem cell (HSCs) transplantation, characterized by donor immune cells attacking recipient tissues [32]. The immunopathogenesis of GvHD involves the activation and proliferation of donor T cells in response to host antigen-presenting cells, resulting in a cascade of inflammatory cytokine release and subsequent tissue damage, predominantly affecting the skin, liver, and gastrointestinal tract [32,33]. Current therapeutic strategies primarily rely on broad immunosuppression using corticosteroids and calcineurin inhibitors; however, these treatments are often limited by incomplete efficacy, systemic immunosuppression, and increased susceptibility to infections and relapse [32,33]. MSCs have emerged as a promising therapeutic modality for GvHD due to their immunomodulatory properties, including the ability to suppress T cell proliferation and promote regulatory T cell expansion [34]. Nonetheless, the clinical utility of MSC-based therapy in GvHD is hindered by challenges such as limited homing efficiency to inflamed tissues, transient engraftment, and variable therapeutic responses [35].

CAR-expressing MSCs may enhance tissue-specific homing and antigen-dependent activation, thereby potentiating their immunosuppressive capacity and improving therapeutic outcomes in GvHD [22,25]. Accordingly, Sirpilla and colleagues designed MSCs that express CAR, which target E-cadherin (CAR^Ecad^-MSCs), and investigated their efficacy in the treatment of GvHD [36]. To generate these modified cells, adipose tissue-derived MSCs were transduced with a CAR construct comprising scFv specific for human/canine cross-reactive E-cadherin, linked to a CD28ζ signaling domain [36]. The CD28 domain was selected for its capacity to activate downstream immunosuppressive pathways in MSCs, thereby enhancing their immunoregulatory functions within inflammatory microenvironments [3]. E-cadherin was chosen as the target antigen due to its critical role in maintaining epithelial barrier integrity [37]. This calcium-dependent cell–cell adhesion molecule plays a key role in regulating epithelial permeability. During inflammatory conditions, functional loss of E-cadherin increases epithelial permeability, facilitating leukocyte transmigration, microbial invasion, and the amplification of local inflammatory responses [37].

In vitro assays demonstrated that CAR^Ecad^-MSCs, upon stimulation with soluble E-cadherin, significantly suppressed T-cell proliferation by more than 70% compared to unstimulated MSCs [36]. This immunosuppressive effect was associated with up-regulation of inhibitory receptors such as CTLA-4 and elevated secretion of the anti-inflammatory IL-10 [36]. These findings suggest that CAR^Ecad^-MSCs can effectively modulate immune responses through antigen-specific activation mechanisms [36].

**Figure 2:**
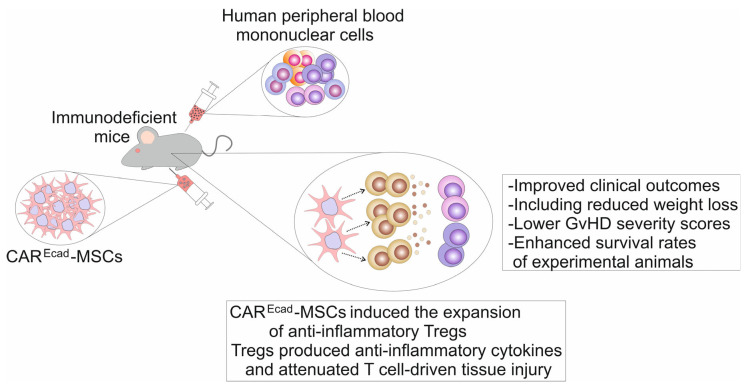
Therapeutic potential of CAR-MSCs in the treatment of GvHD. CAR^Ecad^-MSCs, which were designed to recognize and bind to E-cadherin, efficiently suppressed T cell-driven inflammation in the xenograft model of GvHD that was induced by administration of human peripheral blood mononuclear cells in immunodeficient mice. Intraperitoneally injected CAR^Ecad^-MSCs improved clinical outcomes, including reduced weight loss, lower GvHD severity scores, and enhanced survival rates of experimental animals. CAR^Ecad^-MSCs successfully engrafted in Ecad-expressing tissues, induced the expansion of anti-inflammatory Tregs, which produced anti-inflammatory cytokines and attenuated T cell-driven tissue injury.

To validate these findings in vivo, Sirpilla and colleagues compared the efficacy of CAR^Ecad^-MSCs with that of unmodified MSCs in a xenogeneic GvHD model (Figure 2). This model was established by transferring human PBMNCs into immunodeficient mice [36]. The results demonstrated that intraperitoneal administration of CAR^Ecad^-MSCs (1 × 10^6^ cells) led to improved clinical outcomes, including attenuated weight loss, lower GvHD severity scores, and increased survival rates [36]. Mechanistic analyses revealed that CAR^Ecad^-MSCs exhibited enhanced homing to E-cadherin-expressing tissues and promoted the expansion of anti-inflammatory Tregs, which further contributed to their immunosuppressive activity [36]. Notably, CAR^Ecad^-MSCs more effectively induced Treg proliferation and suppressed T cell-mediated GvHD compared to unmodified MSCs [36]. These findings support the hypothesis that CAR^Ecad^-MSCs, through targeted localization and antigen-specific modulation of immune responses, may represent a novel therapeutic strategy for the treatment of GvHD and other autoimmune conditions [38].

Safety evaluations indicated that CAR^Ecad^-MSCs retained their stem cell phenotype and did not exhibit signs of unwanted differentiation [36,38]. In studies involving healthy canines, administration of CAR^Ecad^-MSCs did not result in adverse effects, including weight loss or organ toxicity, further supporting the favorable safety profile of this therapeutic approach [36,38]. Collectively, these findings underscore the potential of CAR^Ecad^-MSCs as a novel and effective strategy for achieving targeted immunosuppression. Further preclinical studies are warranted to confirm their therapeutic efficacy and safety in models of autoimmune and inflammatory diseases, prior to consideration for clinical application [38].

## 5. Therapeutic Potential of CAR-MSCs and Their Exosomes in the Treatment of Neuroinflammatory Diseases

Neuroinflammatory diseases such as multiple sclerosis (MS), Parkinson’s disease (PD), and Alzheimer’s disease (AD) are characterized by chronic inflammation within the central nervous system (CNS), leading to progressive neurodegeneration, demyelination, and cognitive or motor dysfunction [39,40,41]. Although these diseases differ in etiology and clinical presentation, they share common immunopathological features including activation of resident glial cells (microglia and astrocytes), infiltration of immune cells, and dysregulated cytokine and chemokine signaling [40]. In MS, an autoimmune response directed against myelin antigens leads to recruitment of autoreactive T cells (primarily Th1 and Th17), macrophages, and B cells into the CNS, resulting in myelin sheath degradation and axonal injury [40,41]. Similarly, in PD and AD, microglial activation and release of pro-inflammatory cytokines (TNF-α, IL-1β, and IL-6) contribute to dopaminergic neuron loss in the substantia nigra and amyloid-β plaque formation, respectively [40,41]. Chronic neuroinflammation not only accelerates neuronal damage but also impairs repair mechanisms and disrupts the neurovascular unit, further compromising blood-brain barrier (BBB) integrity [39]. These processes create a feed-forward loop of inflammation and degeneration that drives disease progression [39,40,41].

**Figure 3:**
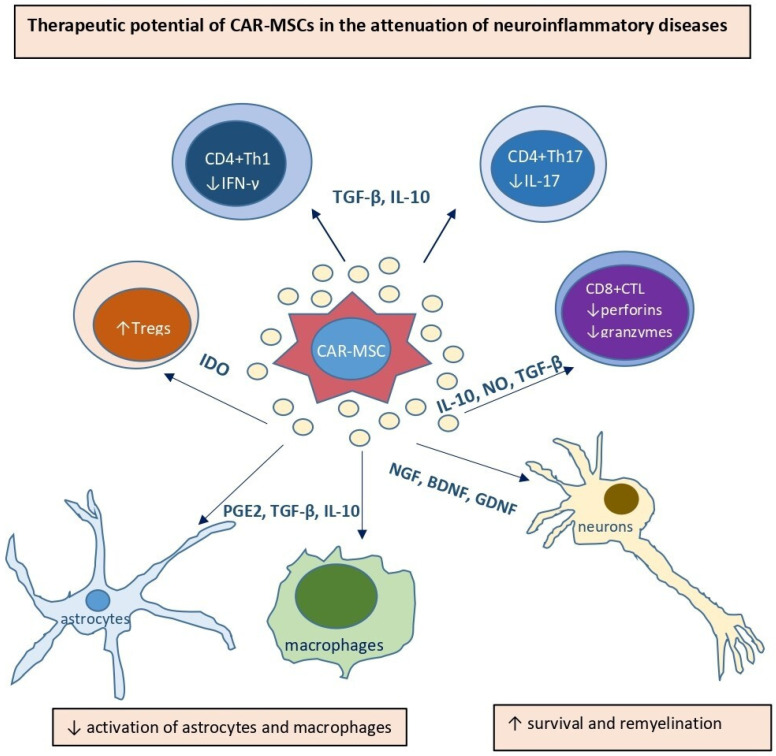
Therapeutic potential of CAR-MSCs in the treatment of neuroinflammatory diseases. CAR-MSCs could (i) suppress production of inflammatory cytokines (IFN-γ, IL-17) in CD4+Th1 and Th17 lymphocytes in IL-10 and TGF-β-dependent manner, (ii) promote expansion of Tregs in IDO-dependent manner, (iii) inhibit secretion of perforins and granzymes in NO, IL-10 and TGF-β-dependent manner, (iv) suppress activation of astrocytes and macrophages, and (v) support survival, plasticity and remyelination of neurons, alleviating progression of autoimmune and inflammatory diseases of central nervous system.

Current therapeutic options are largely symptomatic and do not adequately address the underlying immune dysregulation [41]. In this context, MSCs have gained significant attention for their ability to modulate immune responses and promote neuroregeneration [42]. MSCs exert immunosuppressive effects through secretion of anti-inflammatory cytokines (IL-10, TGF-β) and through cell-to-cell interactions that inhibit the activity of pro-inflammatory T cells, astrocytes, and macrophages while promoting expansion of Tregs [5,42]. Furthermore, MSCs secrete neurotrophic factors (brain-derived neurotrophic factor (BDNF), nerve growth factor (NGF), and glial cell-derived neurotrophic factor (GDNF)), which support neuronal survival, synaptic plasticity, and remyelination (Figure 3) [42]. Preclinical and early clinical studies have demonstrated that MSCs can ameliorate neuroinflammation, reduce lesion size, and improve neurological outcomes in animal models of MS, PD, and AD [42]. However, major limitations such as low efficiency of MSC homing to inflamed CNS regions, transient therapeutic effects, and variable responses among patients restrict their broader clinical applicability [42]. Overcoming these challenges requires novel strategies to enhance the targeting, persistence, and functionality of MSCs within the diseased CNS environment [42].

CARs can be tailored to recognize CNS-specific antigens or markers of neuroinflammation, such as VCAM-1, ICAM-1, or misfolded protein aggregates (amyloid-β in AD or α-synuclein in PD), allowing selective homing of CAR-expressing cells to inflamed regions [22,25]. Accordingly, upon binding to their targets, CAR-MSCs may exhibit enhanced secretion of anti-inflammatory cytokines, neurotrophic factors, and immunoregulatory exosomes in a localized, antigen-dependent manner. This targeted activation minimizes systemic immunosuppression and maximizes therapeutic efficacy at the site of inflammation [22,25].

Despite enhanced targeting capabilities, CAR-MSCs face significant challenges in reaching inflamed or demyelinated regions within the CNS due to their limited capacity to cross the BBB [22]. Exosomes derived from CAR-MSCs (CAR-MSC-Exos) can be engineered to express peptides that facilitate their transport across the BBB via receptor-mediated transcytosis, targeting receptors such as transferrin or insulin-like growth factor receptors, which are highly expressed on brain endothelial cells [43]. Additionally, due to their nanoscale size (typically 30–150 nm), lipid bilayer composition, and intrinsic biocompatibility, CAR-MSC-Exos represent a versatile and efficient vehicle to bypass the restrictive BBB, enabling targeted delivery of bioactive factors derived from CAR-MSCs to brain-infiltrating immune cells and injured neurons, thereby modulating their viability and function [22,43,44,45].

CAR-MSC-Exos can be designed to carry immunosuppressive cytokines such as TGF-β and IL-10, as well as immunoregulatory microRNAs (miR-21, miR-146a, and miR-155), which target key signaling molecules in inflammatory T cells, including the transcription factors STAT1 and nuclear factor kappa B (NF-κB) [22,43,44,45]. Consequently, CAR-MSC-Exos-derived miRNAs may downregulate the differentiation of pro-inflammatory Th1 and Th17 cells, which orchestrate pathogenic immune responses in neuroinflammation, while concurrently promoting the induction and expansion of immunosuppressive Tregs via FoxP3 up-regulation. Furthermore, CAR-MSC-Exos may express programmed death ligand 1 (PD-L1) and Galectin-1 on their surface, contributing to T cell exhaustion or apoptosis through programmed death receptor 1 (PD-1) and CD45-mediated signaling pathways [22,43,44,45].

In addition to effects on T cells, CAR-MSC-Exos may modulate the phenotype and function of innate immune cells within inflamed CNS tissue. They can influence microglial activation, promoting a shift from the pro-inflammatory M1-like phenotype to the anti-inflammatory M2-like state [22,43]. This phenotypic transition is mediated by CAR-MSC-Exos-derived IL-4, IL-10, and heat shock protein 70 (HSP70), which inhibit the production of neurotoxic factors such as TNF-α, IL-1β, and nitric oxide (NO) [22,46]. Additionally, miR-124 and miR-223 contained within CAR-MSC-Exos suppress NF-κB signaling in microglia and macrophages, thereby reducing local inflammation and fostering a neuroprotective environment in injured CNS regions [22,46].

Importantly, CAR engineering can further enhance the specificity and potency of MSC-Exos [22,43]. By targeting CNS-specific inflammatory markers, CAR-MSC-Exos preferentially localize to inflamed brain tissue, facilitating site-directed cargo delivery. This targeted approach minimizes off-target effects and maximizes local immunomodulation [46]. Such localized activity supports remyelination by promoting oligodendrocyte progenitor cell function, and it preserves neuronal architecture through delivery of neuroprotective factors (VEGF, BDNF, and insulin-like growth factor 1 (IGF-1)), which are derived from CAR-MSC-Exos [22,46]. Moreover, the utilization of CAR-MSC-Exos as a cell-free therapeutic modality circumvents several limitations associated with MSC transplantation, including poor cell survival, unpredictable biodistribution, and potential immunogenicity. These features position CAR-MSC-Exos as a particularly promising candidate for the treatment of neuroinflammatory disorders, where continuous immune modulation and neuroprotection are critical [22,46]. Therefore, rigorous preclinical and clinical investigations are warranted to validate these promising findings and establish the therapeutic efficacy of CAR-MSC-Exos in neuroinflammatory disease management.

## 6. The Use of CAR-Expressing MSCs in Clinical Settings: Current Challenges and Future Perspectives

The development and application of CAR-MSCs for the treatment of inflammatory and autoimmune diseases is a rapidly advancing field, and it faces several critical challenges that must be addressed to realize its full clinical potential [22,23,24]. One of the primary obstacles is the heterogeneity of target antigen expression across different immune cell subsets [25]. Additionally, identifying antigens that are specific to pathogenic immune cells but absent from normal tissues remains difficult [24]. Accordingly, a major challenge in the development of CAR-MSC-based therapies for inflammatory and autoimmune diseases lies in the absence of disease-specific target antigens. Unlike malignant cells, which frequently express tumor-associated or neoantigens that are minimally present in healthy tissues, the molecular targets implicated in autoimmunity, such as CD19, ICAM-1, VCAM-1, or E-cadherin, are also constitutively or inducibly expressed in various non-pathological contexts. The immunomodulatory mechanisms of CAR-MSCs, which are designed to be activated in an antigen-dependent manner, could therefore be inadvertently triggered in healthy tissues, potentially leading to undesirable systemic immunosuppression, tissue dysfunction, or impaired regenerative processes. Additionally, compared to CAR-T cells, which are typically employed to mediate cytotoxicity and whose effects can be closely monitored and terminated using suicide switches, CAR-MSCs pose a distinct and potentially greater risk due to their immunoregulatory function, persistence, and tropism for sites of inflammation. As such, the identification of tissue- or context-specific antigenic signatures remains a critical prerequisite for the safe and targeted deployment of CAR-MSCs in autoimmune and inflammatory disease settings [47].

Despite promising preclinical findings, significant questions remain regarding the efficacy of CAR-MSCs, largely due to the complex and multifaceted nature of MSC-dependent immunomodulation. MSCs exert their therapeutic effects primarily through paracrine signaling, secreting a wide array of cytokines and chemokines that influence both innate and adaptive immune responses. These mechanisms are highly context-dependent and influenced by the surrounding microenvironment, making it difficult to delineate a single, dominant pathway of action. Although CARs enable MSCs to recognize specific antigens and potentially localize to sites of inflammation with greater precision, it remains unclear how antigen engagement directly modulates the diverse immunoregulatory outputs of MSCs. Whether CAR activation enhances, redirects, or interferes with existing MSC functions has yet to be fully elucidated. Moreover, the relative contribution of individual immunosuppressive mediators, such as IDO, PGE2, TGF-β, or IL-10, to CAR-MSC-induced immunomodulation remains incompletely defined. This mechanistic uncertainty poses challenges for optimizing CAR design, predicting therapeutic outcomes, and establishing reliable potency assays. Therefore, comprehensive functional studies are needed to dissect the downstream signaling events and effector pathways triggered by CAR engagement in MSCs to fully understand their mechanism of action and therapeutic potential [4,22].

Another significant challenge lies in ensuring the safety and functional stability of CAR-MSCs within the host [22]. MSCs can be influenced by the local microenvironment, which may alter their behavior post-administration [2]. Elevated levels of inflammatory cytokines and hypoxia might affect CAR expression, MSC survival, or therapeutic payload production [24]. Moreover, while MSCs are known for their immunomodulatory properties and low immunogenicity, the introduction of CAR constructs and therapeutic genes may trigger immune responses or reduce their immune-evasive capabilities [8,22]. There is also the risk of uncontrolled MSC proliferation or unwanted differentiation, especially in long-term applications, which necessitates the development of safety switches or controlled expression systems [18]. Additionally, the genetic modification of MSCs to express CARs can lead to unintended immunogenic consequences, including upregulation of human leukocyte antigen (HLA) molecules. Viral transduction and CAR expression may activate innate stress or inflammatory signaling pathways within MSCs, resulting in increased expression of HLA class I and class II molecules. This phenotypic shift may compromise the immune-evasive properties traditionally attributed to MSCs, particularly in allogeneic settings. Elevated HLA expression enhances the visibility of CAR-MSCs to host T cells, thereby increasing the likelihood of immune recognition, cytotoxicity, and rejection. Such immune-mediated clearance can not only reduce the persistence and efficacy of CAR-MSCs but also provoke inflammatory responses that may negate their intended therapeutic benefits. Consequently, careful immunophenotypic characterization of CAR-MSCs post-transduction is essential, and strategies to mitigate alloreactivity, such as transient CAR expression and HLA silencing, may be necessary to enable safe and effective allogeneic administration [7,48].

Importantly, the generation of CAR-engineering of MSCs to express CARs introduces a potential risk of tumorigenicity that warrants careful evaluation [22]. CAR transduction is commonly performed using integrating viral vectors, such as lentiviruses or retroviruses, which randomly insert transgenes into the host genome. This random integration carries a risk of insertional mutagenesis, whereby disruption of tumor suppressor loci or activation of proto-oncogenes may initiate oncogenic transformation. This concern is particularly pertinent in the context of MSCs, given their intrinsic proliferative potential and capacity for long-term engraftment in vivo. Additionally, constitutive or antigen-dependent activation of CAR signaling may dysregulate intracellular signaling networks, thereby perturbing cellular homeostasis and favoring aberrant proliferation or resistance to apoptosis. Moreover, extensive ex vivo expansion of CAR-engineered MSCs during manufacturing can lead to replicative stress, chromosomal instability, and the accumulation of genetic alterations, further amplifying the risk of malignant transformation. In light of these concerns, rigorous preclinical safety assessments, including cytogenetic analysis, comprehensive oncogene and tumor suppressor profiling, soft agar colony formation assays, and long-term in vivo tumorigenicity testing, are imperative to ensure the genomic integrity and biosafety of CAR-MSCs prior to their clinical deployment [22].

Manufacturing and standardization of CAR-MSCs pose additional hurdles [22,23,24]. Efficient and reproducible methods for genetic modification, expansion, and quality control of CAR-MSCs are essential for their translation into clinical-grade therapies [22]. Although non-viral gene delivery methods and advanced genome editing technologies, such as CRISPR/Cas9 or transposon systems, offer safer and potentially more precise alternatives to viral vectors, these approaches still require significant optimization to achieve high efficiency and regulatory compliance. In addition to genetic modification strategies, heterogeneity among MSCs derived from different tissue sources can result in functional variability that affects therapeutic efficacy and reproducibility. This underscores the need for standardized source selection criteria, harmonized cell culture protocols, and validated potency assays that accurately reflect CAR-MSC function in disease-relevant contexts [22].

The clinical-grade production of CAR-MSCs involves a series of complex logistical steps that must be tightly coordinated to ensure product quality, consistency, and regulatory compliance. Key challenges include the sourcing of high-quality MSCs from well-characterized donors or tissue banks, followed by efficient and reproducible genetic modification. The entire process must be conducted in a GMP-compliant facility equipped with appropriate infrastructure for sterile processing, closed-system culture expansion, and real-time monitoring of critical parameters. CAR-MSCs require extensive quality control testing at multiple stages, including assessments of transduction efficiency, viability, sterility, identity, potency, and genomic stability. Batch-to-batch consistency is essential and must be demonstrated through validated assays. In addition, cryopreservation protocols must be optimized to preserve cell functionality during storage and distribution, ensuring that the product remains viable and effective upon thawing at the clinical site. The logistics of scale-up are further complicated by the need to balance manufacturing capacity with individualized or small-batch production strategies, particularly in autologous settings. Finally, coordination with regulatory agencies, clinical investigators, and logistics partners is required for timely delivery, product traceability, and adherence to chain-of-custody and chain-of-identity requirements throughout the clinical trial process [49].

Regulatory considerations surrounding CAR-MSCs are particularly complex due to their status as both cell and gene therapy products [49]. Regulatory agencies such as the U.S. Food and Drug Administration (FDA) and the European Medicines Agency (EMA) require comprehensive characterization of genetically modified cells, including verification of transgene integration sites, assessment of replication-competent viruses, long-term genomic stability, and detailed immunophenotyping. Additional quality control measures, such as sterility testing, endotoxin levels, and functional assays for CAR-mediated activation, must be rigorously implemented and validated. Furthermore, the high cost associated with the production, quality control, and regulatory documentation of CAR-MSCs represents a significant barrier to broad clinical adoption and necessitates strategies to improve manufacturing efficiency and reduce cost-of-goods. Advances in synthetic biology may help to overcome some of these challenges. The incorporation of inducible CAR systems, logic-gated constructs, and safety switches could enhance the precision, control, and safety of CAR-MSC therapies by restricting activity to disease-specific environments or by allowing temporal modulation of CAR signaling [22]. Moreover, engineering CAR-MSCs to express imaging reporters or tracking tags may facilitate non-invasive, real-time monitoring of their biodistribution and persistence in vivo, which is critical for evaluating therapeutic responses and optimizing dosing regimens [22,24].

For the target, cell-specific treatment of inflammatory and autoimmune diseases, CAR-MSC-Exos could be used. These nano-sized vesicles are able to deliver CAR-MSC-sourced immunosuppressive factors selectively to inflamed tissues, injured parenchymal cells, and auto-reactive lymphocytes, offering a localized and safer alternative to systemic immunosuppression [22,24]. Importantly, CAR-MSCs offer a significant advantage over unmodified MSCs in the generation of Exos, as the presence of CAR enables selective and antigen-dependent functional activation [43]. CAR-MSC-Exos can carry enhanced concentrations of immunomodulatory cytokines and regulatory microRNAs following CAR engagement, thereby increasing the specificity and potency of their therapeutic effects. Unlike conventional MSC-Exos, which exert broad and nonspecific immunosuppressive functions, CAR-MSC-Exos can be tailored to recognize and respond to disease-associated antigens, facilitating targeted delivery of bioactive cargos to inflamed or diseased tissues. This targeted activation reduces off-target effects and minimizes systemic immunosuppression. Furthermore, CAR-MSC-Exos retain the favorable properties of Exos-based therapies, including nanoscale size, low immunogenicity, and ability to cross biological barriers while offering enhanced disease specificity through CAR-mediated antigen recognition [43].

## 7. Conclusions

CAR-MSCs represent a versatile and innovative platform with significant therapeutic potential for the treatment of inflammatory and autoimmune diseases (Table 1) [22,23,24,25,31,36,38]. By combining the inherent inflammation-homing abilities of MSCs with the antigen-specific targeting capacity of CARs, this approach enables localized and sustained delivery of therapeutic agents directly into the sites of injury and inflammation [23,24]. In this way, CAR-MSCs could deliver immunomodulatory factors to inflamed tissues, efficiently restoring immune tolerance while minimizing systemic side effects [22]. Despite these perspectives, several challenges still limit clinical application of CAR-MSCs, including antigen specificity, safety, immune compatibility, and scalable manufacturing (Table 2) [22,24]. Therefore, CAR-MSC-based therapies are currently still in the preclinical stage, with no registered human trials to date. Continued research is needed to optimize CAR design, improve control over MSC behavior in vivo, and ensure consistent therapeutic performance across disease models and patient populations [23,24,25]. Emerging technologies such as inducible CAR systems, logic-gated circuits, and genome-editing tools are poised to overcome these obstacles, paving the way for the design and implementation of next-generation CAR-MSC-based therapies for the treatment of inflammatory and malignant diseases [22,23,24].

## Figures and Tables

**Table 1:** Preclinical evidence supporting the therapeutic efficacy of CAR-MSCs.

Disease Model	Source of MSCs	CAR Design	CAR Target	Molecular Mechanisms of CAR-MSCs	Therapeutic Effects	Therapeutic Potential	Ref.
SLE	Human MSCs (unspecified tissue source)	CD19-specific scFv fused to IFN-γ receptor intracellular domain	CD19	Antigen-specific activation of the JAK2/STAT1 pathway; Enhanced IDO expression	Suppressed activation of CD19^+^ B cells	Precision immunotherapy of SLE and other B cell-mediated autoimmune diseases	[31]
Xenogeneic GvHD mouse model	Human AT- MSCs	scFv specific for human/canine E-cadherin fused to CD28ζ signaling domain	E-cadherin	Increased expansion of Tregs; Enhanced suppression of ongoing inflammation	Improved homing of MSCs in inflamed tissues	Precision immunotherapy of GvHD	[36,38]
Alzheimer’s disease, Parkinson’s disease, multiple sclerosis	Human MSC-Exos	scFv specific for CNS inflammation markers; engineered to cross BBB	VCAM-1, ICAM-1, amyloid-β, α-synuclein	Enhanced delivery of IL-10, TGF-β, miR-21, miR-146a, miR-12 in inflamed brains	Suppression of Th1/Th17 cell-driven neuroinflammation; Increased generation of M2 microglia	Cell-free, site-specific immunotherapy of neuroinflammatory diseases	[43,44,45,46]

**Table 2:** Key technical, regulatory, and clinical challenges for CAR-MSC translation.

Category	Specific Challenges	Implications	Ref.
Technical Issues	Lack of disease-specific antigens; Off-target activation in healthy tissues; Complex and undefined MSC immunomodulatory mechanisms; Heterogeneity of MSCs from different sources;	Risk of systemic immunosuppression or tissue damage; Unpredictable efficacy; Variable therapeutic response and immune rejection in allogeneic settings	[22]
Safety Considerations	Insertional mutagenesis due to viral vectors; Chromosomal instability during expansion; Altered MSC behavior in inflammatory environments	Need for comprehensive preclinical safety testing; Requirement for safety switches and controlled CAR expression	[47]
Manufacturing	Complex GMP-compliant production; Need for standardization across tissue sources; Batch-to-batch variability; Cryopreservation logistics	High production cost; Need for validated potency and identity assays; Difficulties in scalability for clinical use	[49]
Regulatory Barriers	Combined classification as cell and gene therapy product; Extensive quality control; Compliance with vector safety regulations	Lengthy and costly regulatory pathways; Requirement for harmonized international guidelines; Delays in clinical implementation	[49]
Clinical Uncertainties	Undefined mechanism of action upon CAR engagement; Lack of robust biomarkers to track efficacy	Hinders clinical trial design and interpretation; Complicates dose selection and patient stratification	[23]
Monitoring and Control	No established in vivo monitoring tools; Difficulty in predicting biodistribution and persistence	Limits safety and efficacy assessment in clinical trials; Necessitates development of real-time imaging tools	[49]
CAR-MSC-Exosome Strategy	Requires optimization of Exos purification and cargo loading; Regulatory uncertainty for Exos-based products	Safer alternative to cell-based therapy; Promising platform for targeted delivery of immunomodulatory factors	[43]

## Data Availability

The data that are discussed in this article are presented in the cited studies.
